# Prognostic value of the blood urea nitrogen-to-albumin ratio for 28-day mortality in septic intensive care unit patients: a large-scale retrospective cohort study using the Medical Information Mart for Intensive Care IV database

**DOI:** 10.1590/1806-9282.20260593

**Published:** 2026-10-12

**Authors:** Jiatong Hou, Jiaqi Cheng, Wentao Liu, Yuefu Wang

**Affiliations:** 1 Capital Medical University, Beijing Shijitan Hospital, Department of Surgical Intensive Care Unit – Beijing, China.; 2 Capital Medical University, Beijing Shijitan Hospital, Department of Anesthesiology – Beijing, China.

**Keywords:** Blood urea nitrogen, Albumin, Prognosis, Sepsis, Mortality, Intensive care units

## Abstract

**OBJECTIVE:**

Sepsis is a life-threatening condition characterized by dysregulated host responses to infection and high mortality in intensive care units. Existing prognostic models are often complex or resource-intensive. The blood urea nitrogen-to-albumin ratio, reflecting renal function, metabolic stress, and nutritional status, has emerged as a potential prognostic biomarker. The aim of this study was to evaluate the predictive value of blood urea nitrogen-to-albumin ratio for 28-day mortality in septic intensive care unit patients.

**METHODS:**

This retrospective cohort study utilized data from the Medical Information Mart for Intensive Care IV (version 2.0) database. Adult patients meeting Sepsis-3 criteria were included. Suspected infection was defined by antibiotic administration and microbiological culture within 24 h. Demographic, clinical, and laboratory data were collected, and blood urea nitrogen-to-albumin ratio was calculated using values obtained within the first 24 h of intensive care unit admission. Receiver operating characteristic curve analysis, Kaplan-Meier survival analysis, and multivariate Cox regression were performed.

**RESULTS:**

A total of 6,940 patients were included, with a 28-day mortality rate of 21.6%. Blood urea nitrogen-to-albumin ratio demonstrated superior predictive performance (area under the curve (AUC)=0.647, 95%CI 0.631–0.664) compared with serum urea (AUC=0.634) and albumin (AUC=0.428), and also outperformed the Sequential Organ Failure Assessment score (AUC=0.613). The optimal cutoff value was 7.6. Patients with blood urea nitrogen-to-albumin ratio ≥7.6 had significantly higher mortality than those with lower values (p<0.001). Elevated blood urea nitrogen-to-albumin ratio remained an independent predictor of mortality after adjustment.

**CONCLUSION:**

Blood urea nitrogen-to-albumin ratio is a simple, objective, and readily available biomarker associated with 28-day mortality in septic intensive care unit patients and may contribute to risk stratification when used alongside other clinical parameters.

## INTRODUCTION

Sepsis, characterized by a dysregulated systemic inflammatory response to microbial invasion, remains a prevalent and life-threatening condition in the intensive care unit (ICU). Approximately one-third of critically ill patients develop sepsis, which can lead to multiple organ dysfunction and increased mortality^1^. Despite advances in organ support and sepsis management, morbidity and mortality remain unacceptably high, making prognostic evaluation a central focus in critical care medicine. Although various biomarkers and scoring systems are used to predict outcomes, many are complex, require multiple parameters, and are often costly or not readily accessible^2^. Therefore, identifying a simple, reliable, and easily obtainable prognostic indicator is of substantial clinical importance.

Blood urea nitrogen (BUN), the end product of protein metabolism synthesized in the liver and excreted by the kidneys, reflects metabolic and renal function. In sepsis, enhanced catabolism and a high risk of acute kidney injury (AKI) frequently lead to elevated BUN levels^3^. BUN is influenced by several pathophysiological factors, including renal hypoperfusion, reduced cardiac output, dehydration, and neurohumoral activation^4^, and has been identified as an independent predictor of prognosis in critically ill patients^5^.

Albumin, which accounts for more than 50% of plasma proteins and is synthesized by hepatocytes, plays a key role in maintaining oncotic pressure and physiological homeostasis. It also serves as an indicator of nutritional status. Hypoalbuminemia is strongly associated with adverse outcomes, including increased mortality, morbidity, and prolonged ICU stay^6^. During infection and systemic inflammation, accelerated proteolysis and increased capillary permeability contribute to decreased albumin levels^5^.

The BUN-to-albumin ratio (BAR), integrating both BUN and albumin, has recently emerged as a novel prognostic biomarker. It reflects both catabolic stress and inflammatory/nutritional status, and has demonstrated predictive value in various conditions, including lung cancer, gastrointestinal bleeding, and community-acquired pneumonia. Recent studies by Cai et al.^7^ and Wang et al.^8^ using the Medical Information Mart for Intensive Care IV (MIMIC-IV) database reported that BAR is independently associated with mortality in septic patients. However, further validation in large and independent cohorts is still required.

Accordingly, the present study aimed to evaluate the prognostic value of BAR for 28-day mortality in septic ICU patients using the MIMIC-IV database. By comparing its performance with individual markers (BUN and albumin) and the Sequential Organ Failure Assessment (SOFA) score, we sought to determine whether BAR could serve as a simple, accessible, and effective prognostic tool in clinical practice.

## METHODS

### Data source

Data were obtained from the MIMIC-IV (version 2.0), a large, publicly available database developed by the Massachusetts Institute of Technology. This database contains comprehensive clinical information, including demographics, vital signs, laboratory results, treatments, and outcomes for patients admitted to ICUs between 2008 and 2019. All patient data were de-identified to ensure privacy protection, and informed consent was therefore waived. The study was conducted with authorized access to the database (Certificate No. 50988851).

### Study population

Adult patients (≥18 years) diagnosed with sepsis according to the Sepsis-3 criteria were included. Sepsis was defined as suspected or confirmed infection accompanied by a SOFA score ≥2 within the first 24 h of ICU admission. Suspected infection was identified based on the administration of intravenous antibiotics and microbiological culture sampling within a 24-h window, following established MIMIC-based algorithms.

The initial cohort comprised 10,578 septic patients. Exclusion criteria included: (1) ICU stay <24 h; (2) pre-existing chronic kidney disease, liver cirrhosis, or malignancy; and (3) missing BUN or albumin data within the first 24 h. After screening, 6,940 patients were included and stratified into survival and non-survival groups based on 28-day outcomes.

### Data extraction and variables

Data were extracted using Structured Query Language (SQL) queries implemented through the MIMIC-IV code repository. Only the first ICU admission was considered. Extracted variables included demographic characteristics (age and sex), vital signs (heart rate, blood pressure, respiratory rate, oxygen saturation), laboratory parameters (e.g., blood cell counts, serum electrolytes, creatinine, and glucose), and comorbidities identified via International Classification of Diseases, Ninth Revision (ICD-9)/10 codes.

The SOFA score was calculated using the worst values recorded within the first 24 h of ICU admission, including PaO2/FiO2 ratio, platelet count, bilirubin, mean arterial pressure or vasopressor use, Glasgow Coma Scale score, and serum creatinine or urine output.

The BAR was calculated as BUN (mg/dL) divided by serum albumin (g/dL), using laboratory measurements obtained within 24 h of ICU admission.

### Statistical analysis

Statistical analyses were performed using R software (version 4.2.1) and Statistical Package for the Social Sciences (SPSS) (version 26.0). The primary outcome was 28-day mortality. Continuous variables were expressed as median [interquartile range (IQR)] and compared using a t-test or Mann-Whitney U test; categorical variables were analyzed using the chi-square test. Receiver operating characteristic (ROC) curves were used to assess predictive performance, and the optimal BAR cutoff was determined by the Youden index. Internal validation was performed using 1,000 bootstrap resampling iterations to assess the stability of the area under the curve (AUC) estimates and to correct for potential overfitting. Kaplan-Meier analysis with log-rank test evaluated survival differences. Multivariate Cox regression identified independent predictors, reporting adjusted hazard ratios (HRs) with 95%CIs. Missing data were handled using complete-case analysis, and the proportion of missing values for each variable was reported. A two-tailed p<0.05 was considered statistically significant.

## RESULTS

### Extent of missing data

The proportions of missing data for each variable are summarized in Table 1. The proportion of missing values ranged from 0% (for age and gender) to 8.54% (for peripheral oxygen saturation, SO_2_), with all variables showing a relatively low level of missingness. Complete-case analysis was therefore considered appropriate for the primary analysis.

**Table 1:** Missing data proportions for each variable.

Variable	Missing (n)	Missing (%)
BUN	1,543	5.73
Albumin	1,234	4.58
SOFA score	1,876	6.96
Heart rate	2,108	7.82
Mean blood pressure	1,987	7.38
Temperature	1,234	4.58
SO_2_	2,300	8.54
Age	0	0.00
Gender	0	0.00
Hypertension	2,108	7.82
CHD	2,108	7.82
Diabetes	2,108	7.82
AKI	1,876	6.96
CRRT	1,987	7.38
Ventilation	1,543	5.73

Missing percentages were calculated based on the initial cohort of 26,941 patients. BUN: blood urea nitrogen; SOFA: Sequential Organ Failure Assessment; AKI: acute kidney injury; CRRT: continuous renal replacement therapy; CHD, coronary heart disease.

### Baseline demographic and clinical characteristics

In the MIMIC-IV dataset, comprising 26,941 sepsis cases as per Sepsis-3, we concentrated on patients who were ≥18 years old and admitted to the ICU for the first time. The cohort was reduced to 6,940 patients after applying the abovementioned exclusion criteria and ensuring that necessary clinical data (e.g., BUN level, albumin level, SOFA score) were available for analysis. The detailed screening process is shown in Figure 1. Table 2 delineates the baseline characteristics of both the 28-day survival group and the non-survival group. Of these, 3,056 (44%) were female, and 3,884 (56%) were male. The median age of the patient cohort was 65 years (interquartile range: 53 to 77). The overall mortality rate within 28 days was recorded at 21.6%. Comparative analysis revealed notable distinctions between the 28-day survival group and the non-survival group. The non-survival group exhibited advanced age (p<0.001) and presented with higher heart rate (HR), SOFA scores, and simplified acute physiology scores (SAPS II), lactate level, vasopressor use, and organ support requirement, while manifesting lower mean blood pressure (MBP), SO_2_, and temperature (p<0.001). The median BAR value was significantly higher in non-survivors compared with survivors (14.5 [IQR: 9.2–22.0] vs. 6.8 [IQR: 3.8–11.5], p<0.001). Additionally, the non-survival group displayed a significantly higher incidence of AKI and continuous renal replacement therapy (CRRT) compared with the survival group (p<0.001). Regarding infection source, pulmonary infection was more prevalent in the non-survival group (48.0 vs. 39.1%, p<0.001).

**Figure 1:**
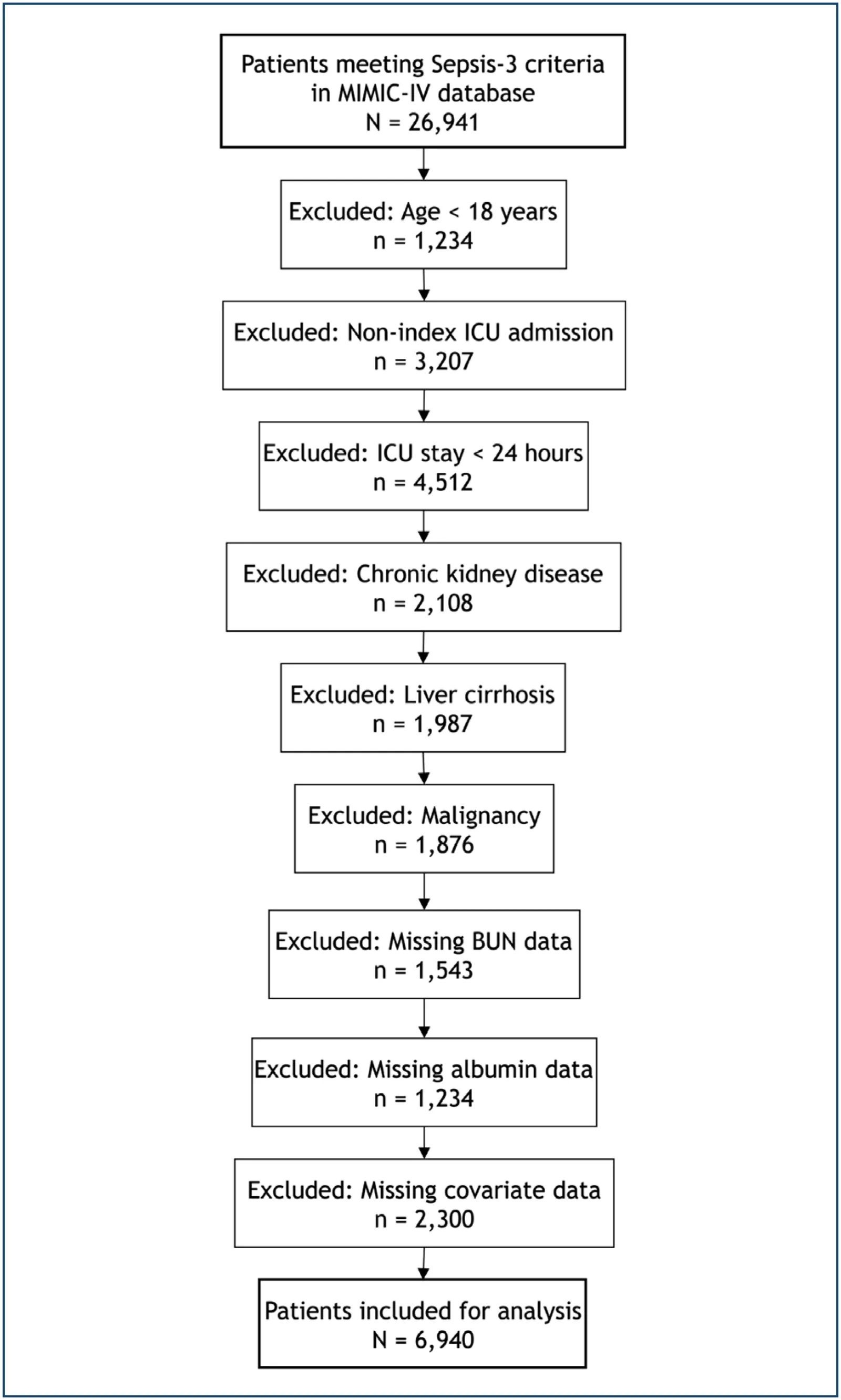
STROBE-compliant participant flow diagram. This flowchart illustrates the stepwise exclusion process of septic patients from the Medical Information Mart for Intensive Care IV database. Starting with 26,941 patients meeting Sepsis-3 criteria, patients were sequentially excluded due to age <18 years, non-index intensive care unit admission, intensive care unit stay <24 h, chronic kidney disease, liver cirrhosis, malignancy, missing blood urea nitrogen, missing albumin, and missing covariate data, resulting in a final cohort of 6,940 patients for analysis.

**Table 2:** Comparison of baseline characteristics between 28-day survival and non-survival groups.

Variable	Total (n=6,940)	Non-survival group (n=1,500)	Survival group (n=5,440)	p
Age (year)	65 (53, 77)	72 (61, 81)	64 (52, 76)	<0.001
Gender (%) female	3,056 (44)	660 (44)	2,396 (44.1)	0.816
Gender (%) male	3,884 (56)	840 (56)	3,044 (55.9)	
Race (%) White	4,277 (61.6)	880 (58.7)	3,397 (62.5)	<0.001
Race (%) others	2,663 (38.4)	620 (41.3)	2,043 (37.5)	
HR (beat/min)	88.2 (76.3, 100.7)	95.0 (85.0, 105.0)	86.0 (75.0, 98.0)	<0.001
MBP (mmHg)	75.6 (69.6, 83.1)	70.0 (65.0, 75.0)	76.0 (70.0, 83.0)	<0.001
Temperature (℃)	36.9 (36.6, 37.3)	36.7 (36.3, 37.1)	36.9 (36.6, 37.3)	<0.001
SO_2_ (%)	97.2 (95.7, 98.6)	96.0 (94.5, 97.5)	97.3 (95.8, 98.6)	<0.001
Lactate (mmol/L)	2.3 (1.5, 4.1)	4.2 (2.6, 6.8)	2.0 (1.4, 3.2)	<0.001
Hypertension (%) yes	721 (10.7)	158 (10.5)	563 (10.7)	0.885
Hypertension (%) no	6,198 (89.3)	1,342 (89.5)	4,856 (89.3)	
CHD (%) yes	368 (5.3)	100 (6.7)	268 (4.9)	0.252
CHD (%) no	6,572 (94.7)	1,400 (93.3)	5,172 (95.1)	
Diabetes (%) yes	347 (5.4)	92 (6.1)	255 (4.7)	0.914
Diabetes (%) no	6,566 (94.6)	1,408 (93.9)	5,158 (95.3)	
SOFA	3 (2, 5)	5 (3, 7)	3 (2, 4)	<0.001
SAPS II	39 (31, 50)	45 (36, 58)	37 (29, 46)	<0.001
Vasopressor use (%) yes	2,186 (31.5)	980 (65.3)	1,206 (22.2)	<0.001
Vasopressor use (%) no	4,754 (68.5)	520 (34.7)	4,234 (77.8)	
Infection source (%) pulmonary	2,846 (41.0)	720 (48.0)	2,126 (39.1)	<0.001
Infection source (%) abdominal	1,526 (22.0)	285 (19.0)	1,241 (22.8)	
Infection source (%) other	2,568 (37.0)	495 (33.0)	2,073 (38.1)	
AKI (%) yes	788 (11.4)	250 (16.7)	538 (9.9)	<0.001
AKI (%) no	6,152 (88.6)	1,250 (83.3)	4,902 (90.1)	
CRRT (%) yes	815 (11.7)	275 (18.3)	540 (9.9)	<0.001
CRRT (%) no	6,125 (88.3)	1,225 (81.7)	4,900 (90.1)	
Ventilation (%) yes	4,048 (58.3)	1,100 (73.3)	2,948 (54.2)	<0.001
Ventilation (%) no	2,892 (41.7)	400 (26.7)	2,492 (45.8)	
Organ support (%) yes	4,356 (62.8)	1,280 (85.3)	3,076 (56.5)	<0.001
Organ support (%) no	2,584 (37.2)	220 (14.7)	2,364 (43.5)	

HR: heart rate; MBP: mean blood pressure; SO_2_: blood oxygen saturation; SAPS II: Simplified Acute Physiology Score II; AKI: acute kidney injury; CRRT: continuous renal replacement therapy; SAPS II: simplified acute physiology scores; CHD, coronary heart disease. Units—HR (beats/min), MBP (mmHg), temperature (°C), SO_2_ (%), age (years).

### Multivariate Cox regression analysis

After adjusting for age, sex, comorbidities [hypertension, diabetes, and coronary heart disease (CHD)], and SOFA score, SAPS II, lactate, vasopressor use, AKI, CRRT, mechanical ventilation, and infection source, multivariate Cox proportional hazards regression analysis revealed that BAR ≥7.6 was independently associated with an increased risk of 28-day mortality (adjusted HR 1.45, 95%CI 1.22–1.72, p<0.001). Other significant predictors of 28-day mortality included age (adjusted HR 1.03, 95%CI 1.02–1.04, p<0.001), SOFA score (adjusted HR 1.12, 95%CI 1.09–1.15, p<0.001), and lactate (adjusted HR 1.08, 95%CI 1.04–1.12, p<0.001), and vasopressor use (adjusted HR 1.35, 95%CI 1.15–1.58, p<0.001). When BAR was analyzed as a continuous variable, each one-unit increase in BAR was associated with a 3.2% increase in the risk of 28-day mortality after adjusting for all confounders (adjusted HR 1.032, 95%CI 1.018–1.046, p<0.001). These findings indicate that BAR, along with other clinical factors, plays a critical role in predicting 28-day mortality.

### Receiver operating characteristic curve analysis

ROC curves for the prediction of all-cause mortality in septic patients were plotted for BAR, BUN, albumin, and SOFA, as depicted in Figure 2. The AUC for BAR was notably higher at 0.647 compared with BUN (AUC, 0.634), albumin (AUC, 0.428), and SOFA (AUC, 0.613). This discrepancy underscores the superior predictive efficacy of BAR. Moreover, the optimal critical value for BAR was identified as 7.6, with a sensitivity of 0.683 and a specificity of 0.55. To assess the stability of the model, internal validation was performed using 1,000 bootstrap resampling iterations. The bootstrap-corrected AUC for BAR was 0.641 (95%CI 0.625–0.658), consistent with the original estimate, indicating good internal validity of the predictive model.

**Figure 2:**
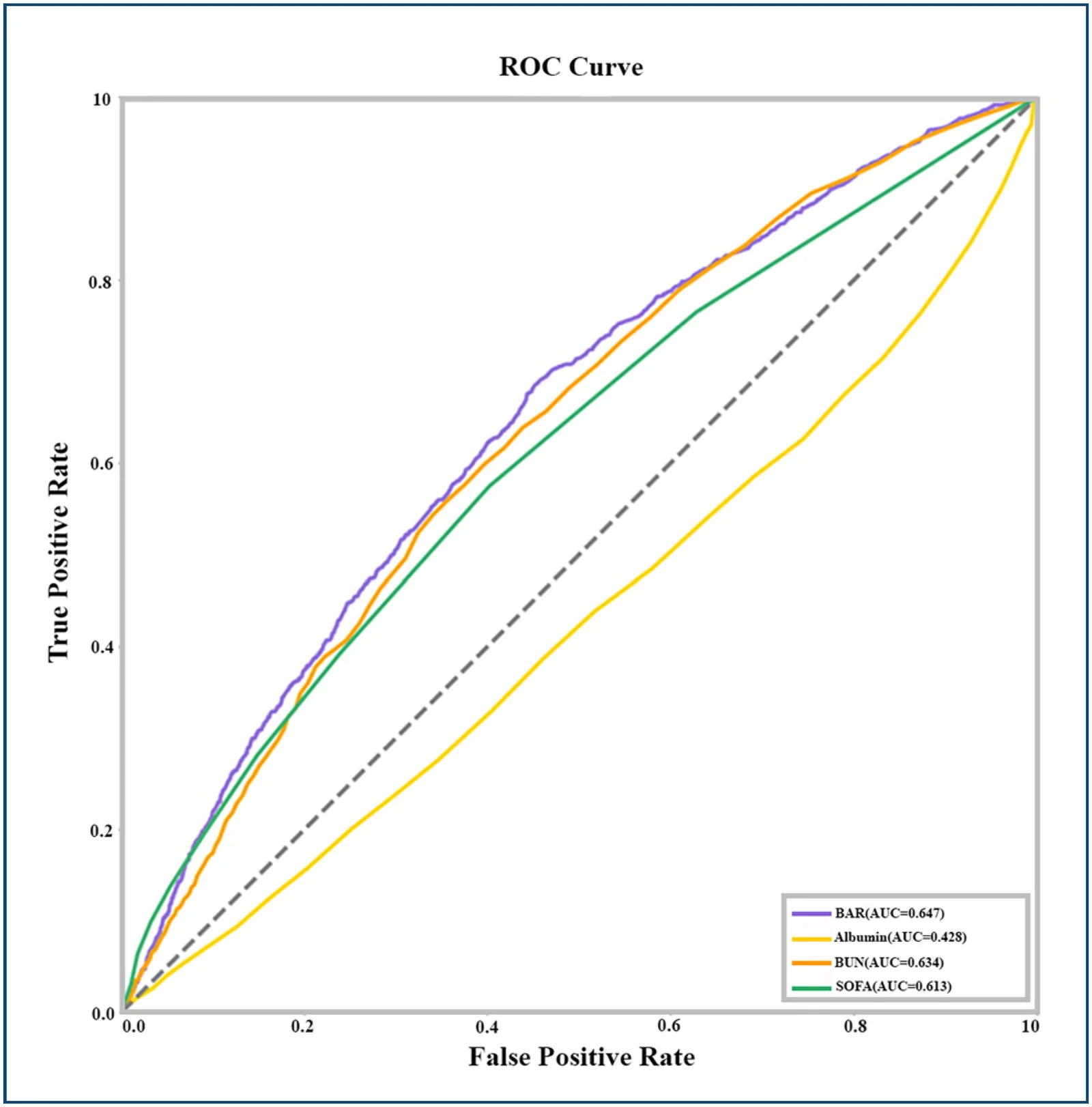
Receiver operating characteristic curves of the blood urea nitrogen-to-albumin ratio, blood urea nitrogen, serum albumin, and the Sequential Organ Failure Assessment score for predicting 28-day mortality in septic intensive care unit patients. The receiver operating characteristic curves compare the predictive performance of blood urea nitrogen-to-albumin ratio (purple solid line), blood urea nitrogen (orange solid line), Sequential Organ Failure Assessment score (green solid line), and serum albumin (yellow solid line). Blood urea nitrogen-to-albumin ratio exhibited the highest area under the curve (AUC=0.647), demonstrating its superior prognostic accuracy for 28-day mortality over the individual markers and the conventional multi-parameter scoring system.

### Kaplan-Meier survival curve

According to the best critical value, patients were divided into high BAR group (BAR ≥7.6, n=3,436) and low BAR group (BAR<7.6, n=3,504). Among these, 1,250 patients (36.4%) in the high BAR group and 250 patients (7.1%) in the low BAR group died within 28 days. The Kaplan-Meier survival curve is illustrated in Figure 3. The results indicated that patients in the high BAR group had a significantly lower survival rate than those in the low BAR group (log-rank p<0.001).

**Figure 3:**
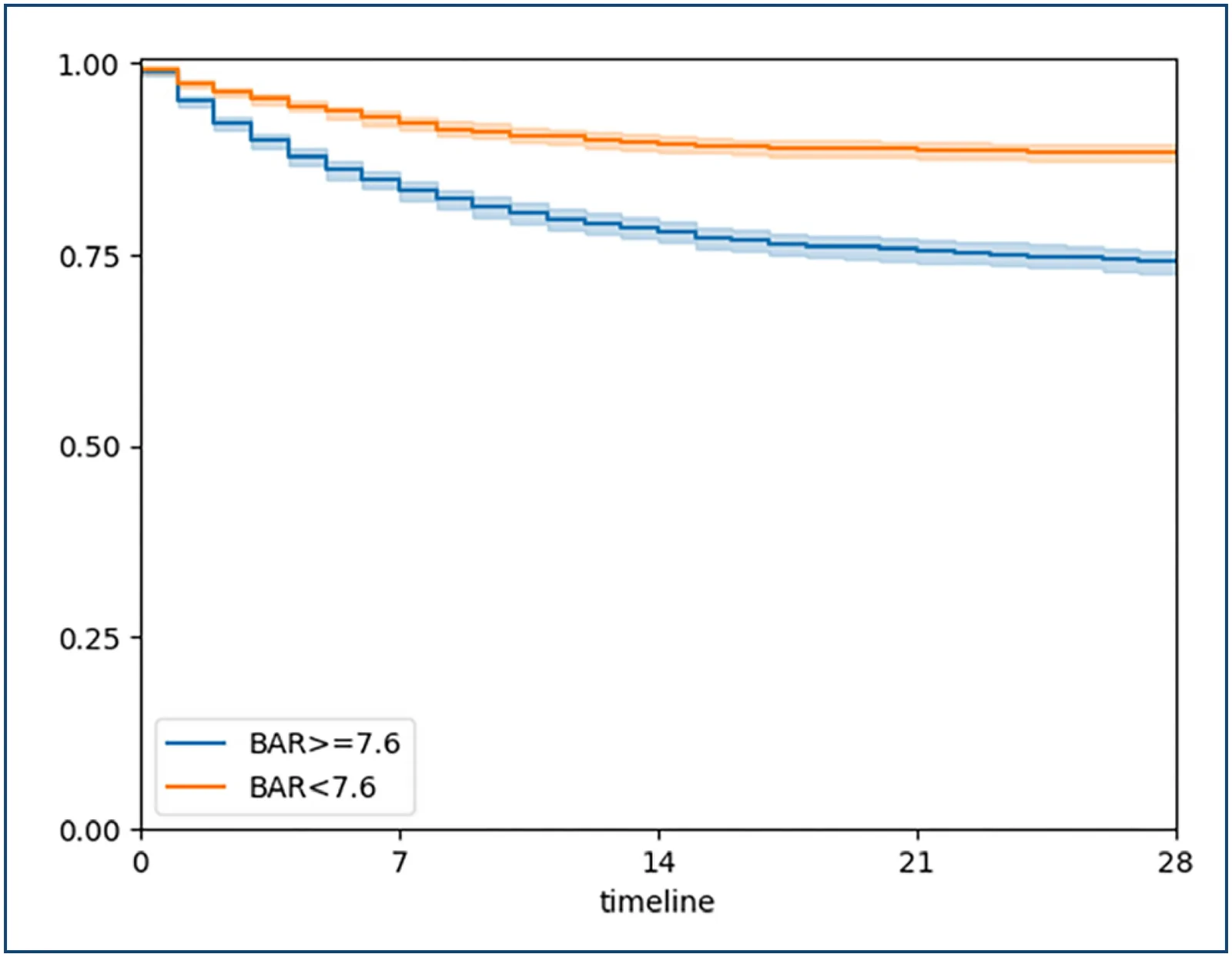
Kaplan-Meier survival analysis curves for all-cause mortality within 28 days of hospital admission. This figure presents the Kaplan-Meier survival analysis based on the optimal critical value of blood urea nitrogen-to-albumin ratio (7.6). Patients are categorized into high blood urea nitrogen-to-albumin ratio (BAR ≥7.6) and low blood urea nitrogen-to-albumin ratio (BAR <7.6) groups, and the survival curves depict the cumulative survival rates over the 28-day period. The results show a significantly higher survival rate in the high blood urea nitrogen-to-albumin ratio group compared with the low blood urea nitrogen-to-albumin ratio group (p<0.001), emphasizing the prognostic value of blood urea nitrogen-to-albumin ratio in predicting short-term mortality.

## DISCUSSION

The present study, based on data from the MIMIC-IV (version 2.0) database, included 6,940 septic patients and demonstrated a 28-day mortality rate of 18.7%. The BAR showed superior predictive performance for mortality (AUC=0.647) compared with serum urea (AUC=0.634) and serum albumin (AUC=0.428), and also outperformed the SOFA score (AUC=0.613). The optimal cutoff value for BAR was identified as 7.6, and Kaplan-Meier analysis revealed significantly higher mortality among patients with BAR ≥7.6. These findings suggest that BAR may serve as a useful adjunctive marker for risk assessment in septic ICU patients, though its modest discriminative ability indicates that it should be interpreted in conjunction with other clinical parameters.

Sepsis remains a major cause of morbidity and mortality in critically ill patients, characterized by a dysregulated host response to infection leading to life-threatening organ dysfunction^8^. Despite continuous improvements in supportive care and therapeutic strategies, the burden of sepsis remains substantial. Therefore, identifying simple and reliable prognostic biomarkers is essential for early risk stratification and timely clinical intervention.

BUN is widely recognized as an indicator of renal function, metabolic status, and protein catabolism^9^. In critically ill patients, systemic stress responses promote protein breakdown, leading to increased BUN production. In addition, enhanced renal tubular reabsorption further contributes to elevated BUN levels^10^. Previous studies have demonstrated that elevated BUN levels at ICU admission, as well as peak BUN levels during hospitalization, are independently associated with increased mortality risk^4,11^. These changes are primarily attributed to intensified protein catabolism and dehydration in critically ill patients^11^.

Albumin, which constitutes more than 50% of plasma proteins, plays a critical role in maintaining oncotic pressure, modulating inflammatory responses, and protecting against ischemia-reperfusion injury^12^. In sepsis, systemic inflammation triggers cytokine release, leading to increased vascular permeability, capillary leakage, and tissue hypoperfusion, all of which contribute to hypoalbuminemia. Reduced albumin levels are consistently associated with poor clinical outcomes and have been identified as independent predictors of mortality in patients with sepsis and septic shock.

Although the SOFA score is widely used for assessing organ dysfunction, its application may be limited in certain clinical settings due to its reliance on multiple parameters. In contrast, BAR integrates two routinely available laboratory indices—BUN and albumin—into a single parameter that simultaneously reflects renal function, metabolic stress, inflammation, and nutritional status. Elevated BUN reflects renal hypoperfusion and catabolic stress, while hypoalbuminemia indicates systemic inflammation and poor nutritional reserves. By combining these complementary dimensions, BAR provides a more comprehensive representation of the pathophysiological alterations in sepsis, which may explain its superior predictive performance compared with individual markers and the SOFA score. Moreover, BUN and albumin measurements are objective, cost-effective, and easily obtainable, enhancing the clinical applicability of BAR.

The present findings are consistent with and extend previous studies by Cai et al.^7^ and Wang et al.^8^, both of which demonstrated that BAR is independently associated with mortality in septic patients using the MIMIC-IV database. Recent studies have further reported the prognostic value of BAR in specific sepsis subpopulations. Han et al. demonstrated that BAR was positively correlated with 28-day mortality in patients with sepsis-induced AKI, with an AUC of 0.639 and an HR of 1.029 per unit increase^13^. Li et al. reported that in patients with sepsis-induced coagulopathy, each 10-unit increase in BAR was associated with a 30% higher hazard of 30-day mortality (adjusted HR 1.18, 95%CI 1.12–1.24)^14^. Compared with prior research, this study offers several methodological strengths. It utilized the updated MIMIC-IV v2.0 database, applied the Sepsis-3 criteria for case identification, and employed rigorous exclusion criteria to minimize confounding factors, such as chronic kidney disease, liver cirrhosis, and malignancy. Furthermore, sepsis was identified using a combination of SOFA scores and standardized definitions of suspected infection, rather than relying solely on administrative coding. Clinically, this study confirmed that BAR outperforms both its individual components and the SOFA score in predicting 28-day mortality in a large cohort of septic ICU patients, thereby enhancing the robustness and generalizability of the findings. Beyond these prior reports, our study provides several novel contributions. First, we evaluated BAR in a broad, unselected septic ICU population (n=6,940), whereas previous studies focused on specific subpopulations. Second, we systematically compared BAR's predictive performance against both its individual components (BUN and albumin) and the SOFA score. Third, we established a clinically applicable optimal cutoff value (7.6) and performed internal validation. Fourth, we integrated multiple clinically relevant confounders including lactate, vasopressor use, AKI, CRRT, mechanical ventilation, and infection source into the multivariable model, providing a more robust adjustment for disease severity.

Nevertheless, several limitations should be acknowledged. First, the relatively modest AUC value (0.647) suggests that BAR, although informative, is not a definitive predictor of mortality and may require integration with other biomarkers or clinical parameters to improve predictive accuracy. Second, the retrospective design introduces potential residual confounding and limits causal inference. Third, BAR was calculated based on a single measurement within the first 24 h of ICU admission, which may not fully capture the dynamic progression of sepsis. Serial monitoring of BAR may provide additional prognostic insights. Fourth, BAR measurements may be influenced by several clinical factors. Exogenous albumin administration, commonly used for volume resuscitation or albumin replacement in septic patients, may directly elevate serum albumin levels and consequently lower BAR values, potentially underestimating mortality risk. Fluid resuscitation, while essential for hemodynamic support, may dilute both BUN and albumin concentrations, introducing variability in BAR measurements that does not necessarily reflect true pathophysiological changes. Moreover, AKI is prevalent in sepsis and directly affects BUN levels through impaired renal excretion, potentially confounding the association between BAR and mortality. While we adjusted for AKI in the multivariable Cox regression model, residual confounding from these dynamic factors cannot be fully excluded. Finally, while complete-case analysis was used to handle missing data, the exclusion of patients with missing data may have introduced selection bias. However, given the low proportion of missing values across all variables, the potential impact of this bias is likely minimal. Future prospective, multicenter studies are warranted to validate these findings across diverse populations and to explore the value of dynamic BAR monitoring in clinical practice.

## CONCLUSION

This study demonstrated that the BAR is a simple, objective, and clinically accessible biomarker associated with 28-day mortality in septic ICU patients. Using data from the MIMIC-IV database, BAR exhibited modest predictive performance, which was superior to serum urea, serum albumin, and the SOFA score, with an optimal cutoff value of 7.6. Patients with BAR ≥7.6 had significantly higher mortality risk. These findings suggest that BAR may contribute to early risk stratification when used alongside other clinical parameters, but its modest discriminative ability underscores the need for caution in clinical application. Further prospective studies are needed to validate these results and to investigate whether combining BAR with other biomarkers can further enhance prognostic accuracy.

## Data Availability

The datasets generated and/or analyzed during the current study are available from the corresponding author upon reasonable request.
